# The Epizootic in Germany

**Published:** 1845-06

**Authors:** 


### ACADEMIE DES SCIENCES.—FEBRUARY.

*The Epizootic in Germany.*—Dr. Schwab, the director of the veterinary school of Munich, has communicated, through M. Royer, an account of the Bohemian epizootic. This epidemic, which he calls *the bovine plague*, declared itself in Gallicia after the passage of oxen from the Russian provinces, and has gradually propagated itself to Moravia and Bohemia. In the former country, from September to December, 1,065 oxen have been attacked, 840 have died, 129 have been destroyed; only 68 have been cured. The disease broke out in Bohemia in September. It appears to be a virulent form of typhus. Every treatment hitherto employed has been inefficacious. The cattle should be destroyed as soon as the existence of the disease has become manifest. It has evidently been propagated in Bohemia by herds coming from infected districts.

